# Remaking critical care: Place, body work and the materialities of care in the COVID intensive care unit

**DOI:** 10.1111/1467-9566.13708

**Published:** 2023-09-13

**Authors:** Catherine M. Montgomery, Annemarie B. Docherty, Sally Humphreys, Corrienne McCulloch, Natalie Pattison, Steve Sturdy

**Affiliations:** 1 Science, Technology & Innovation Studies, University of Edinburgh, Edinburgh, UK; 2 Centre for Biomedicine, Self and Society, University of Edinburgh, Edinburgh, UK; 3 Centre for Medical Informatics, The Usher Institute, University of Edinburgh, Edinburgh, UK; 4 Diagnostics, Anaesthetics, Theatres and Critical Care, NHS Lothian, Edinburgh, UK; 5 Critical Care and Research & Development, West Suffolk NHS Foundation Trust, Suffolk, UK; 6 School of Health and Social Work, University of Hertfordshire, Hertfordshire, UK; 7 Nursing, East and North Hertfordshire NHS Trust, Stevenage, UK

**Keywords:** body work, care, COVID-19, intensive care unit (ICU), materiality, place

## Abstract

In this article, we take forward sociological ways of knowing care-in-practice, in particular work in critical care. To do so, we analyse the experiences of staff working in critical care during the first wave of the COVID-19 pandemic in the UK. This moment of exception throws into sharp relief the ways in which work and place were reconfigured during conditions of pandemic surge, and shows how critical care depends at all times on the co-constitution of place, practices and relations. Our analysis draws on sociological and anthropological work on the material culture of health care and its sensory instantiations. Pursuing this through a study of the experiences of 40 staff across four intensive care units (ICUs) in 2020, we provide an empirical and theoretical elaboration of how place, body work and care are mutually co-constitutive. We argue that the ICU does not exist independently of the constant embodied work of care and place-making which iteratively constitute critical care as a total system of relations.

## Introduction

The actual space that defines an ICU was an essential incubator for the critical care specialty. It enabled us to develop our craft within the security of its four walls.

(Hillman, 2002, p. 594)

The lived body is not just one thing in the world but a way in which the world comes to be.

(Leder, 1992, p. 25)

In this article, we take forward sociological ways of knowing care-in-practice, in particular work in critical care. Our inquiry is based on a study of health-care workers’ experiences of working in critical care during the first wave of the COVID-19 pandemic in the UK. The pandemic disrupted place–care relations on an unprecedented scale, with the Intensive Care Unit (ICU) being a key site of transformation. Unlike some other parts of the hospital, critical care experienced large and rapid increases in activity (Aziz et al., 2020). This was achieved by repurposing other parts of the hospital as ICUs and redeploying staff to work alongside existing critical care colleagues. During the surge of the first wave, intensive care as both activity and place was fundamentally reshaped. This moment of exception provides an opportunity to examine the ICU as a place and the way in which care and place are co-produced through body work (Twigg et al., 2011).

Disruption to usual ways of working in ICU during the pandemic has received sustained sociological attention, deepening understanding of the detrimental psychological effects of the pandemic on health-care workers (De Kock et al., 2021; Hall et al., 2022). In particular, the ways in which the early days of the pandemic reconfigured risk and affect for workers have been well analysed. Drawing on the sociology of emotion and symbolic interactionism respectively, Dowrick et al. (2021) and Rodriquez (2022) show how restrictive visiting policies and changes in the organisation of care led to new forms of emotion management and interactional roles in the ICU. Likewise focusing on affect, Veazey et al. (2021) chart the ‘riskscapes’ health-care workers negotiated when infective risk combined with emotions such as fear to create ‘affective atmos-pheres’ in the emergency room. Emotions in their account are not only individually felt, but, following Ahmed (2004), collective, productive and spatially-defining. By charting how emotions circulated through global news media, digital platforms and local instantiations of care practices, Veazey et al. show how health-care workers came to feel risk, fear and empathy collectively across wide geographic expanses. Their work builds on previous analyses of risk and its role in mediating self-place relations during the pandemic, which have shown how ‘risky work’ spills over between occupational and domestic spheres (Willis & Smallwood, 2021).

The focus on risk and fear is indicative of a broader trend in the COVID-19 literature of fore-grounding internal states of mind in health-care workers’ response to pandemic working. While these studies provide valuable insights, they have often left unanalysed the *embodied* aspects of working practice and the way in which care is *situated* in material environments. This gap was recently highlighted in a critical interpretative synthesis of qualitative studies among health-care workers during the pandemic (Harrison et al., 2022). In their review of 134 articles, Harrison et al. (2022) found that even where studies documented material adaptations to the care environment, ‘critical analysis of the material effects of these adaptations was in most cases limited’ (p. 13). They called for future scholarship to extend interpretive approaches by investigating ‘how care environments adapt (are *re*made) in the face of uncertainty and in times of emergency’ (Ibid). We respond to this call by analysing the (re)constitution of critical care—as place and practice—through the phenomenologically-informed lens of body work (Twigg et al., 2011).

In recent years, there has been an increased focus on the embodied nature of medical work and its sensory dimensions. Informed by phenomenological approaches to the body and relating closely to the concept of medical work as ‘body work’ (Twigg et al., 2011), this scholarship has attended to the way in which clinical knowing is located as much in seeing, touching, hearing and intercorporeal relating as in scientific textbooks and guidelines. Studies have elucidated the way in which surgical training and practice is fundamentally embodied (Prentice, 2007), how doctors learn through their senses (Harris, 2021), how diagnosis is a practical achievement of reading the body (Goodwin, 2010) and the ways in which critical care can be considered ‘craft work’ (Carmel, 2013). Studies focusing on embodiment have brought a new materialist approach to the study of medical work, insisting on the importance of the material environment to the structuring of care (Buse et al., 2018).

Extending the focus on materialities of care, further work considers the *environments* in which health-care is produced (Brown et al., 2020; Martin et al., 2015) and the way place and care are mutually co-constitutive (Ivanova et al., 2016). This sociological literature finds its partner in anthropological studies of hospitals and their spatial relations (Street & Coleman, 2012). Together, these literatures have given us the concepts of care infrastructures (Weiner & Will, 2018) and hospital sensescapes (Bates, 2019), which direct attention to the material, social and sensory work involved in health care. Perhaps counterintuitively, work on the material environments of care has focused on infrastructures—architecture, mundane objects and technologies—while remaining relatively silent about the human bodies which work in these environments. By the same token, research into ‘body work’ has given scant attention to the way in which those both providing and receiving care are situated in particular places. The upshot is a rich conceptual vocabulary for writing about body work and the materialities of care, but a dearth of empirical and theoretical elaboration of how place, body work and care are mutually co-constitutive.

Understanding this nexus of body work, place and the materialities of care is acutely relevant to consider in critical care—both during ordinary times and during the surge situation of COVID-19. As the opening quotation by Hillman (2002) encapsulates, place—specifically ICU— played a vital role in the emergence of critical care as a medical speciality. The first ICUs took shape in Denmark during the 1952 polio epidemic, as a space in which to treat patients requiring prolonged mechanical ventilation. From this grew the idea of ICU as ‘a discrete geographic locale within a hospital or health centre where the sickest patients can be cared for’ (Marshall et al., 2017, p. 272)—an idea subsequently adopted elsewhere, including British hospitals from the mid-1960s (Reynolds & Tansey, 2011). Critical care has since come to be seen as the most high-tech space in the hospital, a ‘data-heavy, complex environment’ deploying ‘high-acuity, time-sensitive interventions’ (Meissen et al., 2022, p. 6). Consequently, the idea of critical care as ‘a speciality defined by a discrete area of the hospital’ (Marshall et al., 2017, p. 271) continues to resonate, to the extent that the terms ‘critical care’ and ‘ICU’ are often used interchangeably. Critical care was never just a place, however. As early as the 1970s, the crucial role of embodied knowledge and practice in critical care was being asserted:

Nothing replaces the knowledge gained by feeling the quality of the pulse, listening to the sound of the systolic and diastolic pressures, the observation of the colour and temperature of the skin and extremities, the type of respiration, and whether or not the jugular vein is engorged.

(Morley & Spark, 1970, p. 851)

More recently, there has been a move to rethink intensive care as ‘a speciality without walls’ (Department of Health, 2000; Marshall et al., 2017), defined by interdisciplinary practices that span the emergency department, hospital wards and follow-up clinics. Understanding the relationship between place, bodies and their practices is thus crucial to thinking about how critical care can continue to be made and remade as a vital part of hospital medicine.

The rapid changes to both environment and staff that took place in the spring of 2020 as ICUs expanded and adapted to the demands of the COVID-19 pandemic provide a unique opportunity to study that relationship. In this article, we take this opportunity to attend to two questions: (1) How did the COVID-19 surge of Spring 2020 lead to a reconfiguration of both place and body work in critical care? (2) What can this tell us about the nature of care in ICU? In answering these questions, we advance sociological theory into the co-constitution of place, body work and care, as well as reflecting on the fundamental question, ‘what is critical care?’

## Methods

Our analysis is based on a study of staff experiences of working in critical care in the UK during the first wave of the COVID-19 pandemic. Between August and September 2020, the research team conducted 40 in-depth interviews with staff from four ICUs across the UK. The study focused on understanding changes in working practice, interaction with patients, technology for family communication, end-of-life care, learning and training, and personal wellbeing and support. Due to COVID restrictions, interviews were conducted via telephone. The embodied and material aspects of ICU care were not the a priori focus of the study, but rather emerged during the analysis, when it became clear that this was a major feature of staff’s accounts. While an obvious disadvantage of using telephone interviews to study body work is the lack of access to the sensory and material world, these data—collected when face-to-face research was not allowed—nonetheless provide a window onto an otherwise inaccessible world.

We recruited from a broad range of ICUs—both teaching and District General Hospitals; tertiary ICUs with general and specialised services and general ICUs; large (more than 30 beds), medium (approximately 20 beds) and small (fewer than five beds). All increased their capacity to cope with the surge of COVID-19 cases in early 2020, either by expanding into previously unoccupied bed spaces, or by using theatre recovery areas. Some units more than doubled their ICU capacity within a very short space of time (days) as part of the national surge expansion. We advertised the study using posters, email and word of mouth. Recruitment was rapid due to the numbers of staff wanting to share their experiences by taking part in the study, mirroring the experience of similar studies conducted at this time (Vindrola-Padros et al., 2020). Participants were sampled according to principles of maximum variation sampling; they included both critical care and redeployed staff (who were moved to critical care during the first pandemic peak) and represented a mix of professional roles: nurses (*n* = 21), doctors (*n* = 9), advanced critical care practitioners (*n* = 1), operating department practitioners (*n* = 3), allied health-care professionals (*n* = 4) and ward clerks (*n* = 2). The sample was diverse with respect to age, gender, seniority and experience working in critical care. Ethical approval was granted by the University of Edinburgh School of Social and Political Science Research Ethics Committee and HRA approval (20/HRA/3270) was obtained.

Staff made time to take part in interviews both during working hours (e.g. on lunch breaks), at the end of a shift, or on their day off. This was in part due to recruitment methods (direct contact, rather than via a manager, where time may have been granted for interviews). Because the interviews took place between the first and second COVID waves, when activity was significantly reduced, scheduling interviews did not pose a problem. Interviews were conducted by CM (sociologist), SH (research nurse/scholar) and NP (clinical professor of nursing) following digitally-recorded informed consent. Other members of the research team included a professor of sociology, critical care consultant and nurse researcher in critical care, with significant combined qualitative research experience. Digitally-recorded, semi-structured interviews lasted between 30 and 80 minutes, and were professionally transcribed.

Data analysis occurred in two phases. Firstly, in order to deliver timely findings to inform ongoing response to the pandemic, the team analysed the data according to the rapid analysis methods proposed by Hamilton (2013) and Taylor et al. (2018); the results of this analysis are published separately (Montgomery et al., 2021). Subsequently, a second phase of analysis took place, during which all interview transcripts were imported into NVivo software for qualitative data analysis and systematically coded using inductively generated codes. These were used to identify themes, with coded sections compared within and across cases to generate higher order generalisations. This iterative process, including coding, memo-writing and interpretation, involved testing the adequacy of categories against the data (constantly turning between codes and data) and then of moving between cases (comparing data to data) (Charmaz, 2014). This article is based on the second phase of analysis.

## Conceptual Approach

Our analysis is closely informed by previous sociological and anthropological work on the material culture of health care and its sensory instantiations. Gardner summarises the kernel of this scholarship when he writes, ‘The relationship between the knowledge-producing clinical body, the patient-body, and clinical space is one of mutual co-constitution. Clinical knowledge emerges from an interaction during which clinical space, the clinician-body, and the patient-body are simultaneously configuring one another’ (Gardner, 2017, p. 128). The ‘knowledge-producing clinical body’ foregrounds the way in which clinicians’ own bodies—in all their material and sensory capacities—are at the centre of what it is to know medically in a health-care environment. Surgery has been a key site of analysis, with several well-known sociological studies exploring touch as a form of knowing alongside technologically-mediated methods (Moreira, 2004, 2006; Pope, 2002; Prentice, 2007). Importantly, Moreira’s (2004) work shows how surgical practice depends on a set of learned and embodied perceptual schemes configured and activated by the surgical space and the material semiotic components which structure it.

Situated, embodied, sensory learning has been the subject of Harris’ work, both on medical education and on the nature of bodily adjustment in moments of mismatch in the clinic (Harris, 2011, 2021). Drawing on Merleau-Ponty’s (1945) theories of the phenomenological body, and putting this into conversation with Bourdieu (2000) and Ingold (1996, 2000), Harris (2011) analyses what happens to habitual embodied practice when things become incongruent. Her focus is on how overseas doctors adjust to new hospital environments. But in drawing attention to the social labour involved in porting clinical skills between one environment and another, her work has much wider relevance, in particular to our own analysis of how health-care professionals adjusted to working in critical care during an unprecedented surge. While Harris suggests we pay more attention to the ‘environmentally-situated nature of medical practice’ (p. 316), however, her own work focuses on how the practitioner adjusts while the environment in which they work remains essentially stable. But what happens when the environment itself is in a state of flux? If, as Ingold (2000, p. 5) suggests, skill is not a ‘technique of the body’ individually-conceived, but rather an entire system of relations ‘within a richly structured environment’ (p. 5), we must ask what happens to this system of relations when that environment is disrupted—as was the case with critical care during the COVID-19 pandemic.

In what follows, we strive to present a symmetrical account of how critical care adapted to that disruption—symmetrical in that it attends both to the environment and to the embodied practitioners within it. This analytic symmetry is essential if we are to make sense of how the pandemic created new forms of inter-corporeal knowing, doing and being in critical care. Our analysis proceeds as follows: firstly, we show the metamorphosis of the ICU into an environment-in-the-making and the ways in which material changes troubled the identity of critical care as learned and skilful habitual practice. Our analysis then looks at the spatial reconfiguration of expertise in COVID ICU, and the impact of the sealed environment on rhythms of work. We show how personal protective equipment (PPE), a key part of the sealed environment, played an important part in shaping body work, including tactile and verbal communication and its affective dimensions. We describe how staff attempted to manage spaces of recovery, decline and death, and we consider how environment and practitioner are not discrete but incorporated one into the other. This takes us beyond the ICU and hospital and into the domestic space of health-care practitioners’ homes, in the ultimate example of the total system of relations which critical care enacts. Our analysis of how critical care adapted to meet the challenges of COVID-19 thus requires that we think, not just about ICU as a place, nor just about how staff changed their practice, but care as a total system of relations between body work, sensory practice and place-making. Critical care and ICU were remade together as simultaneously space and practice, constituted through physical, epistemic and emotional body work.

## Findings

### ‘Not an ICU’: Improvising care and place together

In the four hospitals in our study, anticipation of a surge in patients led to the creation of new places where critical care could be provided over and above usual capacity. The creation of these places happened quickly—typically within a fortnight—and involved assembling both materials and staff. These places acquired various names—pop-up ICU, makeshift ICU, red ICU, COVID ICU, satellite ICU, dirty ICU—which highlighted their distinctive character: compared to the ICUs that staff were used to working in, COVID ICU was seen as makeshift, dirty, untethered, dangerous. This was a result of the rapid reconfiguration of spaces, materials and staff, which was at odds with the usual calm and order of the established ICU:

It’s frustrating because we didn’t have…we were just kind of like a pop-up shop in a way.

(Nurse, Critical Care, Hospital D)

The provisional nature of these spaces required staff to improvise in their efforts to deliver critical care. In hospital C, the COVID ICU was a repurposed recovery area, which staff found stressful to work in, as equipment was unusual (anaesthetic equipment, differing from ICU equipment) and not where it would normally be, and navigating the long space required nurses to run the length of the room:

The main thing was it wasn’t an ICU, it was just a big area that was set up to be an ICU and that was stressful, because your sluice wasn’t where it would normally be, your drugs were away at the other end of the ward, there wasn’t a phone near you, it was away up… You know, it was a big long recovery area that you had to run…

(Nurse, Redeployed, Hospital C)

Similar situations occurred in the other COVID ICUs in our study:

We had taken over another unit, it wasn’t ICU. So, you know, the physical storage capacity within that new unit was really poor and despite the very excellent efforts of members of staff to cobble together something and make it work, it really didn’t work and there was therefore a lot of wasted time on us members of staff having to radio out and ask for pieces of equipment.

(Nurse, Redeployed, Hospital D)

We were being told, right, you must provide… a 400 percent increase in general ICU ventilated beds; and, from my mind, that was bonkers. … we just didn’t have the amount of trained staff and barely had the right amount of equipment to provide that. So, a patient with a thing that pushes air into your lungs regularly and a person who can sort of operate it and watch the patient… that is not ICU, that is a space with a ventilator and somebody next to it. That is not ICU. ICU is so much more than the sum of the individual component parts broken down to their minimum requirements.

(Doctor, Critical Care, Hospital A)

As these quotes illustrate, these places were ‘not ICU’ because the staff and equipment, and the relationships between them, were not *where* they should be, *as* they should be, or were not there at all. For the work of critical care to be accomplished in these new places, additional labour was necessary: things had to be ‘cobbled together’, staff had to cover greater distances, go to find things which should have been at hand. It was not just the spatial and material inadequacies, but the failure to integrate skilled work and spatial order that meant it was ‘not ICU’. As a result, staff had to remake their working environments as best they could, in real-time, while simultaneously enacting care.

They had to do so, moreover, while negotiating new and very material barriers erected to control the spread of infection. How staff experienced, overcame or worked around those barriers serves to highlight the kinds of body work needed to deliver critical care.

### The bubble

A distinctive feature of COVID ICU was its separation from the rest of the hospital by a zippable plastic barrier, creating what staff in some units referred to as ‘the bubble’ (Montgomery et al., 2021). Entry to and exit from the bubble involved a laborious process of donning or doffing PPE, a tangible marker of infectious space which induced anxiety for many staff. The environment inside the bubble was described as hot, noisy and uncomfortable, a departure from the usually calmly organised space of critical care. Staff consistently described it as a stressful environment:

On the other side of the red zone, through the barriers, in PPE it could be incredibly taxing. So, there was a lot of environmental stress…purely working in that environment induced some form of stress in your body.

(Doctor, Critical Care, Hospital A)

Theatre Recovery is like a huge big long room, and there’s no windows, very echoey. And on a normal day, when they’re just recovering patients, and there’s a lot going on, it’s noisy. But when you add in lots of people, lots of machines, and heat, and racket, and then cover your face, and all the stuff we had on, you were shouting to be heard all the time.

(Nurse, Critical Care, Hospital C)

The demarcation of space itself occasioned new ways of working, with implications both for patient care and staff’s sense of inter-professional hierarchy. The primary effect was a change to the rhythm of work. The boundaries around normal ICU are generally quite porous: while nurses provide the backbone of continuous care, doctors and allied health professionals flow in and out (Patel et al., 2022). By contrast, entering the bubble required time to get into PPE and entailed using up sometimes-scarce resources. Consequently, doctors, allied health professionals and managerial staff spoke of going in less frequently for more targeted visits, while nurses said they sometimes missed breaks to stay inside, either because time was short or they felt guilty about using up PPE supplies:

We had to learn to structure our day differently, so just the fact of having a COVID zone where you have to go and wear PPE and then leave again, that limits the time that you spend in the unit… it means that there’s a delay between the decision to go into the unit and getting in and there’s a cost to it because you use PPE every time you go in, so the whole structure of how we arrange our time and how we manage our day changed.

(Doctor, Critical Care, Hospital A)

[The consultants] tended to come in, do a round, and go out again, and we were like, we were told “there’ll be a doctor in there all the time, don’t you worry, there’ll be people all the time”. And half the time, you’re turning round going, “where are the doctors? Bleep them, get them to come in”.

(Nurse, Critical Care, Hospital C)

The rhythms of a shift were, therefore, very different for medical and nursing staff leading to resentment from the latter, several of whom reported feeling trapped inside the bubble with insufficient support. One nurse reported that ‘when you were in work, you were in the, you know, the Red ITU, there was no way you could get away’ (Nurse, Critical Care, Hospital C). In effect, the barriers around the bubble not only created new constraints on work and its rhythms, they also magnified the differences between COVID ICU and the rest of the hospital, and between those inside and outside the bubble, in ways that amplified professional hierarchies and challenged collegiality.

### PPE

PPE, too, created new barriers and constraints, but between bodies rather than places. These barriers likewise disrupted working practices. But in contrast to the bubble, PPE tended to level hierarchies, foreground the importance of cooperation, and bind staff together in solidarity. All staff had to be fit-tested for masks and don PPE before entering COVID ICU, engendering a shared sense of personal vulnerability and a common experience of the physical and emotional challenges it entailed:

People see this as being their barrier between them and coronavirus. In terms of the atmosphere of the unit, I mean the literal atmosphere, changed in that we were all physically bound by PPE.

(Doctor, Critical Care, Hospital B)

PPE affected body work in multiple ways, from interpersonal communication amongst staff to building rapport with patients and providing appropriate care. Because everyone looked the same in PPE, the usual markers of role and expertise were erased, leading to uncertainty over where help could be found and whose competencies could be called on. Building rapport with patients was similarly hampered by the depersonalisation that full PPE entailed, with staff commonly remarking that they must look like spacemen to their patients. Expressing emotion through PPE was difficult and the non-verbal cues that are a key part of communication were lost behind masks, visors and swathes of protective material:

Building the rapport with the patient when they can’t see your face is very, very difficult. These patients are very scared and that was quite challenging actually because they don’t know one person from the other because everybody just looks the same and looks terrifying, …just masked up and gowned up.

(Nurse, Critical Care, Hospital A)

The senses clinicians usually rely on for their work—sight, touch and hearing—were also diminished as a result of PPE. Eyes, ears, mouths and hands were covered by layers of protection: eyes and ears behind goggles, visors or helmets; mouths behind FFP3 masks and hoods; hands inside two or sometimes three pairs of surgical gloves. Some staff, who ‘failed’ fit-tested masks, had to wear negative flow hoods, similar to those used in chemical laboratories, creating further barriers in terms of excessive noise from the whirring of the motors, weight, and anxiety. PPE even created barriers between the different parts of one’s own body. One older nurse described how she was unable to wear reading glasses on the unit, instead relying on a magnifying glass to read drug labels, which made her ‘feel a bit like an old stick’ (Nurse, Redeployed, Hospital A). Another described her distress at providing end-of-life care for one particular patient and how she couldn’t wipe her own tears from her face because of the PPE: ‘I remember just turning round and just, couldn’t see, because I couldn’t wipe my eyes’ (Nurse, Critical Care, Hospital C).

Simple tasks which required fine motor skills, like opening vials and drawing up medications, were hampered by the multiple pairs of gloves that staff wore, making them feel ‘clumsy’, particularly in time-critical situations. Many staff described the challenges of not being able to hear, of constantly having to shout to be heard, of being literally desensitised to the usual cues of patient distress:

You lost a lot of your visual cues and audio cues, so your senses were quite numbed by the PPE. I remember there was one patient, they weren’t intubated and their oxygen saturations were okay… it was only after I came into the bed space that I actually realised the patient was in quite a lot of distress and I hadn’t really noticed. Because I couldn’t hear their breathing … it was only when I got quite near I could see that he had grunting breathing and very tachypnoeic and actually he needed to go on a ventilator. And it’s just a little bit shocking that we hadn’t noticed.

(Doctor, Critical Care, Hospital A)

As this example shows, the ‘body pedagogics’ (Shilling, 2007) that normally enable doctors to read a patient were disrupted—in some cases obliterated—by the layers of protective equipment under and through which they were operating. Adjusting to this new way of being vis-à-vis patients and other staff required constant physical and cognitive effort, which itself was both exhausting and a source of emotional toil.

The difficulties created by absence of accustomed spatial and social order in COVID ICU were, thus, further exacerbated by the erection of material barriers, both around the bubble of COVID ICU and between bodies within the bubble. These barriers reconfigured space and radically altered sensation, disrupting the working practices and interpersonal relations of critical care, and necessitating new ways of working to overcome those disruptions.

### Reconfiguring the spatial organisation of expertise

Yet more disruption was created by the rapid expansion of COVID ICU and the arrival of staff with limited or no previous experience of work in critical care—requiring yet another layer of adaptation. Teamwork is widely recognised as the backbone of ICU. Normally, staff are aware of one another’s specific competencies and work together in close-knit teams with a clear division of labour and norms of communication (Donovan et al., 2018). During the first pandemic surge, an influx of redeployed staff from other parts of the hospital changed the usual dynamics, not only in terms of communication and task-sharing but in terms of the spatial organisation of expertise. While redeployed staff received training, this was necessarily limited and often did not equip them with the skills to safely care for patients on their own in an ICU setting. One senior critical care nurse described how experienced scrub nurses redeployed from theatre were unable to calculate correct fluid balances, make-up drugs or check on infusions, not to mention look after renal dialysis machines. These staff were ‘expected to be a number at the end of the bed looking after really sick people’ but struggled even to record routine observations (Nurse, Critical Care, Hospital C). In order to try and manage this dilution of experienced critical care staff with inexperienced staff, she sought spatial solutions to keep patients safe:

[Y]ou had a line on one side of the wall of patients, and a line on the other side, and you would try meticulously, I would try and put some of my senior staff in the middle of each area, and it would be a triangle effect, that they would look after a ratio, like three to one, which is absolutely crazy.

(Nurse, Critical Care, Hospital C)

Maintaining this triangle of expertise was challenging as staff needed to take breaks and on several occasions were reportedly so traumatised by the work that they did not return. In this situation, the normally ‘controlled’ environment of ICU was replaced by a ‘fraught’ one; the constant effort to create and re-recreate critical care meant that experienced staff had to iteratively adjust their habitual embodied practice.

### Spaces of recovery, decline and death

Another aspect of critical care highlighted by the peculiar circumstances of COVID was practitioners’ concern to manage the aesthetic of ICU, that is, the way it was perceived and felt by the senses. This was seen to be important for two key groups: patients who were improving, and the relatives of patients close to death. Staff were acutely aware of what COVID ICU looked like, both to themselves and their patients:

The conscious patients were terrified, because they knew they had this horrible disease that everyone was scared of…and they could see people dying of that disease all around them by the time they were in the intensive care unit.

(Doctor, Critical Care, Hospital A)

Their descriptions were vivid and nightmarish, focusing on the de-personalisation of staff rendered unrecognisable by PPE ‘spacesuits’, and of patients deformed by disease and the effects of sedation, intubation and proning. Nursing staff in particular actively sought to make adjustments—rearranging beds, screens and curtains—so that recovering patients could not see those who were deteriorating:

[T]hey weren’t great screens in Theatre Recovery, their curtains aren’t brilliant. So you’d have somebody really sick, and then somebody maybe doing bedside sits with the physio, three bed up. And these people could see what’s going on. So we were then trying to move the beds around, so the ones that were getting better couldn’t see the really sick ones. But it was really hard to screen the patients from what else was going on.

(Nurse, Critical Care, Hospital C)

As well as trying to obscure sicker patients from the sight of those who were recovering, staff found various ways to create an aesthetic which would reassure and encourage patients on their path to recovery. This was sometimes static and representational, such as PPE adorned with flowers or pictures, printouts of photos from family members—and sometimes involved staff producing their own spectacle for patients:

[A recently extubated patient] was surrounded by ventilated patients, so he had no one to interact with. And patients typically get ICU delirium, so we were so desperate to try and just interact with him. So…we would dance, you know, little things like that, like every hour we’d…just to try and keep morale up, we’d stop and have a little dance break, you know, or … I would put my phone in a specimen bag and then seal it, and then take it into the bay and put You Tube on for him so he could watch something.

(Nurse, Critical Care, Hospital D)

The fusing here of connection and separation in the account of the nurse’s personal phone shared with the patient in a specimen bag for infection control purposes is illustrative of a wider phenomenology of safety in the ICU. Staff worked creatively with the objects of infection control, expanding their affective connotations from risk avoidance to enablement and social affinity.

Aesthetic concerns also came to the fore when staff faced the challenge of bringing relatives into the hospital space to say goodbye to loved ones who were about to die. ICU is normally porous to families and relatives, who are recognised as a valuable asset and have defined roles in the patient’s recovery (Xyrichis et al., 2021). Very early in the pandemic, this was not possible, but as time passed and staff became more confident in improvising (both with visiting policies and available space), efforts were made to bring dying patients and relatives together in the same place. In Hospital C, staff adapted unused theatres for this purpose, away from the noise and bustle of COVID ICU. In the quote below, the ‘theatre’ assumes both a literal and metaphorical role, being at once a mundane hospital space and a place now charged with the social ritual—or performance—of death:

If the family wanted to come in, people would go out and get them from the front door, bring them to the back door of theatre, and set up a theatre to kind of be as nice as possible. And the anaesthetic room was made into a little relatives’ waiting area. So essentially, we would wheel the person in, on the basic life support that we had them on, and just, it was horrible. Open the anaesthetic room doors, and then two family members, they could go in and be with them, just as they pass away.

(Nurse, Critical Care, Hospital C)

These efforts to manage the aesthetics of end-of-life care took an emotional toll on staff. Driessen et al. (2021) have written about the ‘placing work’ that palliative care staff, patients and relatives undertake to align place and matter during end-of-life care. In COVID ICU, staff likewise under-took placing work, but this was severely circumscribed by pandemic procedures concerning space and visiting rights. In spite of their efforts to make the place of death ‘as nice as possible’, their experience of this as health-care professionals was ‘horrible’. Other research has shown how such difficulties in delivering what they considered good care led many staff to experience ‘moral injury’ (Kok et al., 2023).

The distress staff experienced was not limited to their inability to align place and matter for a good death for patients and their relatives; it extended into the period after a patient died, when porters would usually assume responsibility for moving the body from the ICU to the morgue. During the first pandemic wave, porters were not allowed into the sealed space of the ICU, meaning nurses had to undertake some aspects of this work: ‘we were dealing with loading bodies onto mortuary trolleys, which is not something I’ve ever had to do in my 20 years before’ (Nurse, Redeployed, Hospital A). This nurse, who had worked in critical care for many years before leaving and then being redeployed back during the pandemic, described it as one of the most difficult aspects of patient care; it was an unexpected situation completely outwith her learned embodied practice:

[T]he porters said, “oh, we can’t come in there, we’ll just push the trolley into you” − because they’ve no PPE − and I said to the porter, “which end does the head go at?”, because I didn’t know. I didn’t know if there was a special particular end of the trolley that the head needed to be. And he said, “it doesn’t matter, love, he’s dead”, and I just remember thinking, oh my goodness, this is just, like, way more than I was ever prepared for.

(Nurse, Redeployed, Hospital A)

This layering of aesthetic and affective experience in COVID ICU thus highlights the embodied emotional labour involved in the delivery of critical care. To the work of remaking unfamiliar space, overcoming material barriers and adapting to disrupted working relationships, we must add the challenges of caring for patients’, families’ and health-care professionals’ own feelings.

### Contiguous work-home space

This affective body work did not cease with the end of a shift, or when staff members doffed their PPE and left the confines of the bubble. It continued well beyond COVID ICU, beyond the hospital doors, and into the domestic spaces of health-care workers’ homes. Twigg et al. (2011) describe the persistence of emotions as a fundamental aspect of body work:

The emotional component of body work has thus to be managed as part of the job. It also transcends and permeates boundaries between formal paid employment and the lives beyond, for emotions generated through body work are not easily shed or cast off when the worker leaves the workplace, especially when the workplace is a health and social care setting.

(p. 175)

Our own study not only confirms this carry-over of emotional body work from workplace to home, but shows that, like so much about the experience of delivering critical care, it was amplified by the peculiar circumstances of COVID-19.

During the first UK lockdown, the British public were exhorted to ‘Stay Home, Protect the NHS, Save Lives’ (Anon, 2020). People emptied from public spaces; shops, cafes and restaurants were shut, streets deserted. But while most workplaces were closed and empty, the hospital was open, and ICU busy. For staff coming in to work 12.5 hour shifts, there was a sense of isolation and surreality. The hospital was no longer one workplace among many, but a singular destination for the very sick and those who cared for them. Where staff journeys to and from the hospital had previously been punctuated by visits to social spaces such as cafes and supermarkets, they now became a time of acute and solitary self-awareness. A leitmotif in our data was journeys spent in tears.

This sense of isolation and lack of everyday sociability was all the more acute for those critical care staff who moved out of home in order to protect their families from infection. One nurse described how she moved into a hotel every week to work her hospital shifts, leaving her children with her husband for half the week while she was in COVID ICU:

It was quite a lonely existence. I said goodbye to my children every week, went off, stayed solitary in this hotel, you know? It was just a very solitary existence.

(Nurse, Redeployed, Hospital A)

For those who went home between shifts, meanwhile, domestic life was pervaded by a sense of peril. Other research into health-care workers’ experiences during COVID-19 has documented their fears of bringing the virus home to their families, and conceptualised those fears in terms of risk across the work/home boundary (Willis & Smallwood, 2021). In our own study, participants spoke not only of the emotions they experienced but also of the way these affected everyday acts at home. This included disrupting their usual routines of dressing, showering and laundry; changing how they ate and slept with family members; and intervening in their relations of proximity and affection with loved ones:

During COVID, I came in and undressed in the hallway, put my clothes straight in the washing machine, ran upstairs and showered and disinfected everywhere. And I’m still sleeping in the spare room. I didn’t hug my son for the whole three months…And we self-isolated at the dinner table, so normally we’d all sit together… whereas we spaced ourselves throughout the whole table. And then at home we space ourselves out enough….We haven’t hugged because [my husband’s] at high risk.

(Operating Department Practitioner, Redeployed, Hospital D)

Such accounts were common in our data.

Staff also spoke of their efforts to reassure family members of their safety at work. Just as they sought to choreograph aesthetic space for patients and relatives within COVID ICU itself, so staff improvised visual ways of reassuring their families. Some used television images and newspaper reports as a way to help relatives understand the work they were doing in the ICU. One nurse described how she brought images of herself dressed for work home to her children to reassure them:

I [did] little things like taking a photograph of me in full PPE so that I could take it home to my kids and say “this is all the stuff Mummy wears, I’m really safe from all the bad bugs”.

(Nurse, Redeployed, Hospital A)

Ultimately, the embodied work both of keeping family members safe and of reassuring them about the safety of ICU exerted a high toll on staff, who spoke of the otherwise private acts of familial-preservation they undertook, such as rewriting wills and ensuring guardianship for their children in case of their death. Far from being a sealed space, COVID ICU was a total system of relations, within a richly structured environment that extended from the workplace into the most intimate corners of workers’ lives (Harris, 2011; Ingold, 2000).

## Discussion

In this article, we have drawn on phenomenological studies of health-care work, alongside research in anthropology and sensory studies, to illuminate the experiences of staff working in critical care during the first wave of the COVID-19 pandemic in the UK. At the outset, we posed two questions: (1) How did the COVID-19 surge of Spring 2020 lead to a reconfiguration of both place and body work in critical care? (2) What can this tell us about the nature of care in ICU? Our analysis leads us to the following conclusions.

Firstly, place, practitioner and care are co-produced and interdependent. The imperative to increase ICU capacity by increasing beds and ventilators did not—could not—increase intensive care as it is known by its practitioners. In spite of efforts to create ‘satellite’ ICUs with beds, equipment and staff, participants in this study described the ongoing work required to make and *re*make ICU as patients and personnel, guidelines and PPE supply fluctuated. Rather than simply reproducing a stable, pre-existing environment, staff were constantly engaged in the attempt to create meaningful clinical space—what we earlier refer to as an environment-in-the-making. However, as our analysis shows, the ‘placing work’ (Driessen et al., 2021) required to safely treat patients, humanely encourage recovery and enable a good death for those unable to recover was severely compromised where space, permeability and practitioners were restricted.

Secondly, improvisation and problem-solving are key features of critical care. In an oral history of British intensive care between 1950 and 2000, Ronald Bradley, the first full-time intensive care clinician in the UK, describes how in the late 1950s he crafted ‘an array of measuring kit’ at St Thomas’ Hospital, London:

So I found myself sawing up lengths of steel tubing and making a scaffolding and putting wheels on the bottom of it so that we could take four pressure heads, a set of gas electrodes and an ECG and a recorder on which you could write the pressure records and everything else that came out.

(Reynolds & Tansey, 2011, p. 33)

In his ethnographic study of three UK ICUs in the early 2000s, Carmel (2013) argues that medical practice more broadly is a ‘craft activity’, in the sense of requiring the practical application of knowledge in interaction with the material world. Our own findings confirm these insights, and go further, suggesting that the material world of the ICU does not exist independently of practitioners’ interaction with it, but is created in and through body work. This has important implications for assessing the medical response to COVID-19. At the end of the first year of the pandemic, Surma et al. (2020) berated the critical care community for its failure to implement a learning health-care system, resulting in ‘extraordinary workarounds’ (p. 1907). While our own research did not look at the effectiveness of treatment protocols in critical care, it suggests that workarounds were an outcome of *learning* that is part of everyday ICU and were ‘extraordinary’ because the circumstances were extraordinary. Emphasising a culture of embodied learning does not diminish the profound impacts that the pandemic had on the organisation and experience of work in critical care during COVID-19. Due to the speed and scale at which change was needed, improvisation in this case occurred under duress. However, we would argue that the workarounds Surma et al. (2020) highlight were an indication, not of failure, but of success. Improvisation did not reflect an inherent deficit in critical care competence (despite deficits in PPE and other resources); on the contrary, it was an expression of the embodied experience of being a practitioner in the ICU.

The same insight applies to more future-oriented proposals to implement learning health systems underpinned by data sharing and artificial intelligence (Enticott et al., 2021). As critical care seeks to take advantage of advances in data-driven health care, attention must be given to the embodied and sensory dimensions of practitioners’ work and the total system of socio-material relations which characterises the ICU. Sociologists and anthropologists are well-placed to contribute to an understanding of how new relations are being forged in this context. Design anthropology has been used to understand sensory and embodied experiences, as well as tactile ways of knowing and doing in health-care workplaces (Pink et al., 2014, 2020). The STS and medical anthropology literature more broadly directs us to consider learning as a situated, embodied, sensorial process which is affected by the setting (Harris, 2011, 2021). Recent work on palliative care by Driessen and colleagues draws on well-established theories of intra-action and matter-ing (Barad, 2003; Law, 2004) to trouble the presumed singularity and stability of place and death in health-care management policies. Our own research echoes these studies and demonstrates that intensive care is not a place in any enduring or disembodied sense of the word.

So what, then, is critical care? As our research during the peak of the COVID pandemic shows, critical care is an emergent practice, in which place-making is inherent in the achievement of care. Learned ways of embodying critical care practice cannot be divorced from the constitution of the ICU as place—a place which does not pre-exist the practices which enact patient care on a continuous basis. Challenges to the practical achievement of critical care—whether the spatial delimiting of the ICU, the addition of extra beds beyond a unit’s capacity, or the exclusion of relatives—necessarily have a major impact on body work and professional identity, which in turn informs the creation of the ICU anew. Previous research examining the spatial reassembling of health-care practitioners, patients and technologies (Lehoux et al., 2008), or the practices of clinicians when they relocate to unfamiliar environments (Harris, 2011), has not fully acknowledged the dual and co-dependent activities of place-making and body work. This has been an ongoing feature of critical care since its inception in the 1950s, but became particularly pronounced in pandemic times, and endures in the commitment for critical care to be based on patient acuity regardless of location (Department of Health, 2000). Now, as calls to reconstitute the ICU yet again, this time as a data-driven place, become ever more prominent (Meissen et al., 2022), it is all the more important to appreciate how health-care practitioners incorporate new practices— including data practices—into their existing body work.

## Data Availability

The data that support the findings of this study are available from the corresponding author upon reasonable request.
